# How can we get Iraq- and Afghanistan-deployed US Veterans to participate in health-related research? Findings from a national focus group study

**DOI:** 10.1186/s12874-018-0546-2

**Published:** 2018-08-29

**Authors:** Alyson J. Littman, Gala True, Emily Ashmore, Tracy Wellens, Nicholas L. Smith

**Affiliations:** 1 Seattle Epidemiologic Research and Information Center, Department of Veterans Affairs Puget Sound Health Care System, Seattle, WA USA; 2 Department of Epidemiology, University of Washington School of Public Health, Seattle, WA USA; 3 Seattle-Denver Center of Innovation for Veteran-Centered and Value-Driven Care, Health Services Research and Development, Department of Veterans Affairs Puget Sound Health Care System, Seattle, WA USA; 4 South Central Mental Illness Research Education and Clinical Center, Southeast Louisiana Veterans Healthcare System, New Orleans, Louisiana USA; 5 Section of Population and Community Medicine, Louisiana State University School of Medicine, New Orleans, Louisiana USA; 6 Gnosis Research, Seattle, WA USA; 7 Kaiser Permanente Washington Research Institute, Kaiser Permanente Washington, Seattle, WA USA

**Keywords:** United States, Veterans, Recruitment, Focus groups, Trust, Operation Enduring Freedom, Operation Iraqi Freedom

## Abstract

**Background:**

Research participant recruitment is often fraught with obstacles. Poor response rates can reduce statistical power, threaten both internal and external validity, and increase study costs and duration. Military personnel are socialized to a specific set of laws, norms, traditions, and values; their willingness to participate in research may differ from civilians. The aims of this study were to better understand the views of United States (US) Veterans who served in Operation Enduring Freedom (OEF)/ Operation Iraqi Freedom (OIF) on research and motivators for participating in research to inform recruitment for a planned observational study of respiratory health in OEF/OIF Veterans.

**Methods:**

We conducted 10 focus groups in a purposive sample of OEF/OIF Veterans (*n* = 89) in five US cities in 2015. Key topics included: reasons for participating or declining to participate in health-related research, logistics around study recruitment and conduct, compensation, written materials, and information sharing preferences for study results. Two authors independently coded the data using template analysis.

**Results:**

Participants identified three criteria that motivated a decision to participate in health-related research: 1) adequate compensation, 2) desire to help other Veterans, and 3) significance and relevance of the research topic. For many, *both* sufficient compensation and a sense that the study would help other Veterans were critical. The importance of transparency arose as a key theme; Veterans communicated that vague language about study aims or procedures engendered distrust. Lastly, participants expressed a desire for studies to communicate results of their specific health tests, as well as overall study findings, back to research participants.

**Conclusions:**

OEF/OIF Veterans described trust, transparent communication, and respect as essential characteristics of research in which they would be willing to participate. Additional studies are needed to determine whether our results generalize to other US Veterans; nevertheless, our results highlight precepts that have been reported as important for recruitment in other populations. Researchers may benefit from using community-engaged research methods to seek feedback on recruitment materials and strategies prior to initiating research. For costly studies targeting a large sample (i.e. in the thousands), it may be important to test a variety of recruitment strategies.

**Electronic supplementary material:**

The online version of this article (10.1186/s12874-018-0546-2) contains supplementary material, which is available to authorized users.

## Background

Recruitment of research participants is often fraught with obstacles [1] and is one of the largest costs associated with conducting trials and observational studies [2]. Prior studies have indicated that 65–70% of trials do not meet their original sample size target, and more than half extend the length of the recruitment period to reach targets [3–6]. Failure to enroll the pre-specified sample size is likely to result in insufficient power. Even when sample size goals are met, participation must be independent of the exposure and outcome under study for the research to obtain unbiased results (i.e., high internal validity). High response rates are also important in terms of generalizability (i.e., external validity). Lastly, high response rates are necessary to keep studies on time and budget.

United States (US) military personnel are socialized to a specific set of laws, norms, traditions, and values [7, 8]. Defining characteristics include honor, bravery, personal sacrifice; and commitment to duty, mission, and fellow unit members [7]. Fellow unit members often form strong bonds with each other due to their shared experiences. Authors have noted that active duty service members and Veterans constitute a distinct subculture because of these differences [9]. Furthermore, those who serve in the military are predominantly male--though the number of women serving has substantially increased since 2001-- and more likely to come from the working class than the civilian population [10]. All US Veterans who were discharged from the military under any condition other than ‘dishonorable’ may qualify for Department of Veterans Affairs (VA) benefits; those who were injured during service or fall below a certain income are given the highest priority and may receive healthcare at no charge [11]. Notably, VA reserves the right to reassess disability claims and adjust ratings and benefits for conditions that are not considered permanent, such as posttraumatic stress disorder and back pain. Additionally, unlike US civilians for whom the primary research funding agency (i.e., National Institutes of Health) is separate from their health care provider and insurer, determination of eligibility for benefits, clinical care for Veterans, and much of the research on Veterans’ health are all housed within and funded by the US Department of Veterans Affairs.

Consequently, US Veterans’ willingness to participate in research may differ from civilians, and different approaches to study recruitment may be needed. There is a paucity of research into best practices for recruiting Veterans for research studies [12–17], particularly with respect to those who served in OEF/OIF, research involving observational cohort studies (vs. clinical trials), and for Veterans who are not using VA for their health care. Prior studies in Veterans have noted altruism [17] as an important factor for recruitment whereas difficulty contacting target participants (possibly related to the fact that younger Veterans are highly mobile and military contact information may be outdated) [13], lack of interest [13], and research “burn-out” due to multiple requests [16] were barriers to participation. Bayley et al. hypothesized that participation in their study may have been low due to a low perceived compensation rate ($200 for a full day of assessments) [13].

Prior to the launch of a study of the long-term impacts of open-air waste burning (“burn pits” [18]) on the pulmonary health of military service members, we undertook a formative study using focus group methodology (described in Methods) to maximize the likelihood of successful and timely recruitment. The parent study is investigating associations between land-based deployment and pulmonary health among Veterans who served in OEF/OIF recruited from six metropolitan areas, with a target sample of 4800 participants. Participants attend in-person assessments to measure pulmonary function, height and weight and complete self- and interviewer-administered questionnaires (estimated to take 2–3 h). While the primary outcome (pulmonary function) and exposure (particulate matter) are measured objectively via lung function testing and satellite-based methods, respectively, the investigators were concerned about disproportionate recruitment of individuals who had pulmonary symptoms. Thus, the planned approach (and that tested in the focus groups) was to describe the study with broad aims to elicit interest from a range of Veterans.

The aims of this formative focus group study were to better understand the views of Veterans eligible for the parent study-- US Veterans who served in OEF/OIF -- on research and identify motivators for participating in research studies. The current paper reports findings that generalize to other studies targeting US OEF/OIF Veterans and that may also be relevant to research among Veterans of other eras and non-Veterans.

## Methods

Between September and November 2015, we held 10 focus groups in 5 cities (see Table 1). Cities in four of the five VA regions (Southeast, West, Midwest, and Continental) were selected. We aimed to include 6–12 participants per group, an optimal size to encourage discussion and ensure everyone’s voice is heard [19]. To identify potential participants, we used the Defense Manpower Data Center, which collates personnel and other data for the Department of Defense and includes a complete list of all discharges after 1972 [20].

**Table 1:** Focus group locations, number of attempted contacts per location, and characteristics of each group

Location	Number of individuals contacted	Phone calls needed to fill group?	Enlisted women- only group	Enlisted male-only group	Enlisted mixed sex group	Officer male-only group
Seattle, WA	W: 96	Yes	1	0	0	0
	Enlisted M: 490	Yes	0	1	0	0
Houston, TX	Officer M: 97	Yes	0	0	0	1
	Enlisted M&W: 500	Yes	0	0	1	0
Long Beach, CA	Enlisted M&W: 970	No	0	1	1	0
Minneapolis, MN	Enlisted M&W:471	Yes	0	0	2	0
Atlanta, GA	Officer M: 133	No	0	0	0	1
	W: 500	Yes	1	0	0	0
Total	–	–	2 groups, 15 total	2 groups, 20 total	4 groups, 37 total	2 groups, 17 total

*M* men, *W* women

### Inclusion/exclusion criteria

To be eligible for inclusion, a Veteran had to have served in the US Armed Forces (including Guard or Reserves) between October 1, 2001 and December 31, 2012, had at least one deployment to Iraq, Afghanistan, Kuwait, Qatar, Djibouti, United Arab Emirates, or Kyrgyzstan, and completed their active duty military service as of December 31, 2012. Additionally, we only contacted those who lived within 50 miles of the zip code where the focus groups took place based on address information obtained from a commercial vendor. Exclusion criteria included serving only in the Navy and Coast Guard due to the nature of the parent study, which was focused on land exposures during deployments.

### Recruitment of study participants

A random sample of individuals who were eligible were mailed a packet including an introductory letter inviting them to participate in the study approximately 3–4 weeks before each focus group. The letter including names and phone numbers of investigators and was mailed in a large manila envelope. Non-responders were phoned approximately 1 week later unless the group was already filled or the Veteran notified staff that they were not interested in participating. Two days prior to the focus groups, staff telephoned participants to remind them of the time and location and to answer questions.

### Focus group methodology

In collaboration with the focus group moderator, the investigators and other research staff members developed a focus group guide (Additional file 1). Key topics included: reasons for participating or declining to participate in health-related research, logistics around study recruitment (e.g., preferred mode of contact), acceptable time commitment for surveys and in-person visits, compensation, study logistics (e.g., location), written materials, and sharing information about study results.

Sessions were led by a professional, doctoral-level moderator (TW) who was not a VA employee. To increase the comfort of participants and encourage safe and open dialogue, we held some women-only and enlisted-only focus groups. Some of the enlisted-only groups included only men; mixed-gender groups included at least four women. Focus groups were held in a conference room in a hotel in a central location that was easy to access via car and that had ample, free parking. Total on-site time commitment was approximately 2 h. This included approximately 15 min for informed consent procedures before the focus group, 90 min for the focus group, and approximately 10 min to complete a 7-page survey immediately following the focus group. The survey collected information on demographics, health status, receipt of VA services and/or benefits, military service, and perceptions of the VA and VA research.

To help ground the discussion of recruitment materials, participants were provided with examples of a contact letter and key study procedure information (See Additional file 2). All focus groups were audio recorded. TW summarized findings after each focus group. One team member (AL) attended four of the focus groups and took notes during the sessions. Individuals were compensated $150 (in cash, given on site) for their participation. All study procedures were approved by the VA Central Institutional Review Board.

### Analysis

We analyzed the data using template analysis [21, 22]. The initial template was organized into domains based on focus group questions, and included space for noting majority opinions, exemplar quotes, and outlier responses. Two team members (AL and EA) listened to audio-recordings to evaluate the template for usability and relevance; the template was modified based on initial use and review. After consistency was established, AL and EA independently coded six of the ten focus groups. Exemplar quotes were transcribed verbatim. Summary points were transferred into a matrix (e.g., organized by domain × focus group [23]). Microsoft Word was used for data management. After coding of the six focus groups, notes taken by AL for the remaining 4 and by TW for all 10 were evaluated for consistency with the six templates created by AL and EA. It was not a study objective to examine differences in views and opinions based on gender, officer status or location; thus, findings were analyzed without respect to these characteristics.

## Results

A total of 89 individuals participated in focus groups. On average, participants were 38 years of age (range: 26–66 years), 29% were female, 23% were Hispanic, 55% had a Bachelor’s degree or a professional degree, 21% were officers, and 56% reported their health to be excellent or very good (Table 2). Less than half (46%) used VA health care benefits; over 75% thought that VA benefit services were excellent, very good, or good.

**Table 2:** Characteristics of Veterans who participated in 10 focus groups conducted in 5 US cities (n = 89)

Characteristic	Total	
	N	%
Age (years)		
26–29	14	15.7
30–34	25	28.1
35–44	28	31.5
45–66	22	24.7
Female	26	29.2
Ethnicity (2 missing)		
Not Hispanic, Spanish or Latino	67	70.0
Puerto Rican, Cuban, Spanish, Hispanic, or Latino	20	23.0
Race		
White	50	56.2
Black/African American	21	23.6
All others, mixed, missing	18	20.2
Education		
Technical or trade school, some college or Associate’s degree (all high school graduates or GED)	40	44.9
Bachelor’s degree	32	36.0
Master’s degree, professional, or doctorate degree	17	19.1
Current work status		
Working full time or part time	65	73.0
Unemployed, searching for work	3	3.4
Unemployed, not searching for work, disabled, retired	9	10.1
Student	12	13.5
Pay grade		
Enlisted	70	78.7
Officer including warrant officer	19	21.4
Self-rated health		
Excellent/very good	50	56.2
Good	29	32.6
Fair/poor	10	11.2
Ever use of VA health care benefits		
No	48	53.9
Yes	41	46.1
Current impressions about VA benefit services		
Excellent/very good	34	38.2
Good	33	37.1
Fair/poor	20	22.5
Not sure	2	2.3
Participated in VA health research in the past		
No	65	73.0
Yes	16	18.0
Do not recall	8	9.0
Participated in non-VA health research in the past		
No	72	80.9
Yes	11	12.4
Do not recall	6	6.7

*Abbreviations*: *GED* general equivalency degree (equivalent to a high school graduate), *VA* Department of Veterans Affairs

### Factors affecting participation in research

Table 3 details key factors, comments, and quotes related to factors affecting participation in research. Key findings are briefly highlighted here. The key determinants of participating in health-related research were three-fold: 1) receipt of adequate compensation for participation, 2) *“duty, honor, and doing the right thing” --* a desire to fulfill an obligation to help other Veterans, and 3) perception of the research topic as relevant and important. For many, *both* sufficient compensation and a sense that the study would help other Veterans were critical.

**Table 3:** Key findings related to factors affecting participation in research

Factor	Comments	Quotes
Factors that bolstered their motivation to participate		
Duty, desire to help others	The obligation to help other Veterans was described as one might talk about bonds with a family member	“*If you hit my patriotism and my ability to help others and myself at the same time… [the money] is nice to compensate my time, but for me, that’s not the motivator. It [the compensation] is a motivator, but it is low on my totem pole*.” “I’d [participate] for my *brothers and sisters in arms*.”
Topic seemed important		“*I missed putting my daughter to bed. I’d do it on principle because I believe in it*.”
Financial benefit	Compensation offered as part of participation was a motivator for many and caught their attention.	*“I noticed the $150.”* (on the contact letter)
Personal interest		“*I have had issues that they can’t figure out. Maybe this will help them understand what happened and how to treat it*.”
Legitimacy of research	Study materials made it clear that the study was a legitimate study and not a “scam”	
Factors that detracted from their willingness to participate		
Concerns about study-associated risks	Individuals wanted reassurance that they were not going to be experimented upon	“*If they were going to give me an experimental drug, I wouldn’t do it. I am real skeptical.”*
Concerns about sharing personal information	People were concerned about sharing information about mental health conditions because it would involve disclosing sensitive, personal information with someone who they did not know well and whose job it was to collect information but not provide treatment. People were concerned that information shared could affect their benefits	*“Most research studies are mental health-related and I don’t want to be vulnerable.”* *“Everything is stored in one file so if you don’t get a low score they may take away your benefits.”*

Considerations regarding the relative costs (e.g., inconvenience, time away from work and family) and risks related to privacy and information security, losing VA benefits, and study participation (e.g., experimental drugs) were important potential deterrents to participating in a research study. Before considering participation, Veterans needed assurance that the study was legitimate and not a “*scam*.” Several participants stated that they conducted an internet search of the names and phone numbers of investigators listed in the invitation letter. While not a sufficient reason for participation, interacting with study personnel who were professional, courteous, knowledgeable was also noted as being helpful.

### Initial approach by postal mail using a large envelope followed by phone call is best

Participants generally endorsed the method that was used to recruit them into the focus group study (which involved a mailed letter followed by a phone call) compared with an initial approach via email or phone. Letters delivered via postal mail were preferred because it was easy to recognize military materials and was perceived as more “*official*” than email and less intrusive than a phone call. One participant stated, *“I don’t like phone calls. Especially not survey phone calls.”* Many individuals stated that they opened the letter for the current study because the envelope “*stuck out*”, seemed important and “*official*” (because of the emblem). Legitimacy concerns were heighted with email; words such as “*phishing*” and “*scams*” were used to describe reactions to an initial study approach via email. Overall, Veterans said they “*wouldn’t have paid much attention*” to an email invitation.

Whereas initial contact by phone was not recommended, follow up via phone was acceptable and appreciated by many. The participants noted, however, that they were unlikely to answer the phone, so it was essential that the study recruiter leave a message. One person stated, “*When I see a number and I don’t know it, I won’t answer it. But if you leave a message, I’ll call back.”*

### Introductory letter should contain certain information and wording

During the focus groups, participants were asked to review and provide feedback on an example letter informing them about a study and inviting them to participate (Additional file 2). Table 4 summarizes the findings regarding key issues raised and recommendations about how to address them in written communication. Key overarching themes were related to transparency and trust. Vague language was off-putting. Many participants felt that the letter did not specify *why* the study was being done, which heightened their suspicion. One participant stated, *“Tell us what you are looking for. It feels like it is hidden.”* Another participant added*, “What the hell did I pick up that they know about that I don’t?”* In response to how the letter could be rewritten, one participant said*, “Saying something like, ‘Troops are coming back with respiratory problems; we want to know why’ might be helpful.”* Vague terms were also unpopular. The letter mentioned that the in-person examination will require that patients take a “bronchodilator.” One participant said, “*Bronchodilator? It sounds invasive. Why? What is the reason behind it? If they gave me a valid reason for doing it, I’d be on board*.” Other individuals worried that it was an experimental drug.

**Table 4:** Problems/concerns and recommendations about how to address them in writing or orally to potential participants

Specific problem/concern	Recommendations
Unclear study purpose	To the extent possible, clearly explain why the study is being conducted. Explain how the study will help others (e.g., Veterans)
Letters are too wordy and key information is not easy to extract	Simplify – keep it to Who? What? Where? When? How much time? If individuals can choose the day and time that they come, make that clear. Include sufficient white space in letters. Consider using bullet points.
Individuals are concerned that participation may affect their Department of Veterans Affairs (VA) benefits	State that participation cannot affect their VA benefits. This statement could be bolstered by explaining with whom their information would and would not be shared, and that the research conducted is independent of other VA or governmental entities.
Study appears to be a “scam”	Provide names of researchers. When calling participants, leave a message. Most individuals will not answer a phone number that is unfamiliar to them. Leaving a message can increase legitimacy. Staff phone lines during business hours so that those interested can speak to a study team member.
Vague description of study procedures (e.g., “non-invasive tests”, “bronchodilator”). Vague terms prompted some to be anxious that the drug was experimental and/or that important information was being withheld.	Be specific when appropriate (e.g., provide generic and name-brand drug names – “Albuterol” rather than a “bronchodilator”), so that individuals can better understand study requirements, conduct their own research, including speaking with their doctor about potential risks.
Concerns about inquiries about mental health status, exposure to combat or other stressful situations	Describe the setting for questions/interviews/assessments (individual vs. group). Explain why those questions are being asked, and provide example questions.
Individuals may misunderstand the meaning of being chosen “randomly”	Individuals want to feel special, not “random.” Craft introductory letter to demonstrate that each individual was chosen and that his/her participation is appreciated.
Study-related activities described do not match up with study duration	Provide an agenda so that individuals know that their time will not be wasted.
Inclusion of information (like a consent form) that is for their information and not to be completed	State that materials should be read and not completed. Otherwise, participants may feel that their efforts were a waste of their time since it may need to be done in person.

Individuals generally preferred a less wordy style, as explained by one person, “*People in the military like concise information. Brief*.” Another participant added, “*Keep it to Who? What? When? Where? Why? and How?*” Several participants read the letter that explained that the hypothetical study 2–3-h study visit and could not understand how the activities listed (filling out some questionnaires, “*blowing into a tube*”, and getting height, weight, and pulse measured) could take that long. They assumed that the 2–3 h would involve waiting. However, when the moderator clarified that the 2–3 h would not involve waiting, the feedback we received about the time commitment was generally positive; most participants said that 2–3 h was acceptable provided that they felt that the study team was well-prepared and their time was well spent.

### Logistics for face-to-face study visits that are important

The investigators were concerned that locating study procedures at a VA medical center would dissuade Veterans who do not get their health care from the VA from participating. This concern was not borne out, though a few participants mentioned that they would not want to go to a VA medical center because “*you don’t know what you’re going to walk into in the waiting room*,” referring to the potentially disturbing experience of seeing disabled or sick Veterans. Nevertheless, only one person stated that she would not participate if required to go to a VA. Many saw locating study activities at a VA medical center as an advantage because it was familiar, they knew how to get around, it established legitimacy, and they assumed that they would be taken care of by people who understand Veterans. The cons related to difficulty finding parking, concerns about inefficiencies (the need to wait during their study visit), and “*taking appointments from sicker Vets.*” The third point revealed an important misunderstanding – that some Veterans do not distinguish research from clinical activities. While some found the idea of preferred parking at a VA medical center attractive, others were concerned that they would be taking parking away from Veterans who were older or more disabled than they were.

### Questionnaire content, length, administration

Individuals’ initial response to acceptable questionnaire length was 15–30 min, because with longer questionnaires, “*you get bored*” and “*lose interest*.” Another noted that “*the longer the questionnaire, the less accurate you become. Focus drifts away.*” While for some, compensation would provide motivation to fill out longer/boring questionnaires, others stated that it would need to be clear how the information was relevant. Many expressed a preference for completing a survey on the computer, while a few expressed concerns about data security if done on the web. Others expressed sentiments that implied that they thought that we (VA researchers) should already have the information, because the information was in their Department of Defense or VA record. To illustrate this perspective, one Veteran explained, *“Nothing frustrates me more than having to enter duplicate info. All of that stuff is in the system already. Why are they asking us?*” Participants stated that they would be more willing to complete a longer survey if they had a few weeks to complete the survey, so that they could choose a time to work on it that was convenient for them, spreading out the work over more than one session.

### Compensation for participation

Expectations for compensation for future studies mirrored that offered for participation in the focus group (e.g., $50–75/h, depending on whether individuals included travel time). For others, compensation was not important. One person said, “*If it was considered valuable, I would do it for free*.” Nevertheless, this was a minority opinion and for many, the compensation was important. Generally, participants preferred cash or check on site rather than having to wait for a mailed check because of concerns that the paperwork or the check might be lost in the mail/system. If the compensation could not be given in person at the time of participation, it was important that it be provided within a reasonable amount of time (2–3 weeks). While a debit card with no fees was considered acceptable, a gift card to a specific store was the least preferred mode. Alternative methods proposed by the participants included direct deposit and in-kind compensation, for example priority processing of claims.

### Data sharing

Individuals were interested in obtaining reports of overall study results because it would “*give us the value of what we invested our time in*.” Furthermore, it might increase the likelihood of individuals participating in future studies. One participant explained, “*A lot of research studies don’t follow back up with you and say, ‘this is what we got from the research.’ That would be very helpful. That may make me want to do another one.*” In addition to knowing how the study may have helped others, they wanted information about their own health. Like when they visited their doctor, individuals expected to get a copy of their results so that they could better understand their health status and risk factors. They also expressed a desire to share the study information with their doctor. Participants wanted the report to be easy to understand quickly and to provide basic information like “*Are you in the normal or abnormal range?*” They were also interested in seeing how their measures compared to others who were like them (e.g., in terms of age, service era, deployments, etc.).

## Discussion

Trust, transparency, and respect were themes than ran through our findings on why OEF/OIF US Veterans participate in research and how they wish to be treated as research participants. Singer and Ye concluded that there are three primary reasons why individuals participate in research: 1) altruism/norms of cooperation, 2) egoistic/self-interest (e.g., enjoys surveys, would benefit, interested in learning something new and “the money”, and 3) responses mentioning one or more survey characteristics (e.g., interest in the topic, respect for organizations, length of survey) [24]. While these reasons generally align with those observed in the current study, US Veterans’ military socialization, shared experiences, close bonds with fellow Veterans, and relationships with the VA differentiate them from civilians and may impact their perspective on research participation. Findings from this study reinforce the importance of working to establish trust. Similar to vulnerable populations such as indigenous people [25], other minority populations [26–29], and frail elderly adults [30], due to a history of harassment, assault, or other trauma [7], trust and safety concerns are more prevalent among military Veterans, potentially making recruitment more challenging. For US Veterans to participate, they must perceive that the researchers are trustworthy and that the research is of value. Trust will be enhanced by being transparent -- clearly stating the study’s purpose and intended impact(s) of the research, consistent with previous research in other populations [31]. Vague purpose statements were perceived as disingenuous and diminished trust. Veterans also wanted to be reassured that the research was legitimate, and the research team will be good stewards of their data. Providing information on the study team can facilitate information gathering efforts by potential participants.

Respect is also a core military value [7]; several barriers and facilitators to participation related to displays of respect. To demonstrate respect, researchers should consider providing generous compensation; preparing an agenda for face-to-face study visits; and being efficient, professional, and on time. Also, as observed in prior studies [32–34], US Veterans reported wanting to receive results of study-related tests and study findings. While data sharing may not directly impact willingness to participate in the short term, it demonstrates respect and appreciation for the study participants and might motivate participation in future studies.

Our study yielded other interesting findings that we wish to highlight. Many Veterans misunderstood how the Department of Defense, Veterans Benefits Administration, clinical services managed by the VA, and VA-funded research were connected. Some assumed that they had already signed waivers for data sharing, making it possible for researchers to obtain military records. Conversely, they feared that information given in a research study might be shared with other entities and affect their benefits. Memoranda of understanding regarding data sharing are difficult to understand and often have broad provisions such that misunderstandings are likely [http://citeseerx.ist.psu.edu/viewdoc/download?doi=10.1.1.473.3931&rep=rep1&type=pdf]. Likewise, data sharing clauses in research consent documents are often wide-ranging and provisional. Determining ways to more clearly and simply communicate this information may help to avoid misunderstandings in the future. Furthermore, unlike a prior study [16] that emphasized the value of employing military members as research staff (who have an “insider” perspective), participants responded positively to our staff, none of whom were Veterans.

Lastly, our study revealed that relatively small changes, like using a large envelope instead of a small one may help avoid mail being tossed without being read. We are aware of only one study [35] that examined response rates for a large vs. small envelope; that study found no difference, though the subjects were physicians.

Several limitations should be considered when evaluating our results. First, we were unable to obtain information about perceptions of research among those who did not volunteer to participate in our research study. Nevertheless, most of our study participants had never participated in a health research study, suggesting that we may have included individuals who had previously declined participating in research. It is possible that the generous compensation motivated Veterans who might have been otherwise unlikely to participate. In support of this hypothesis, some studies indicate that higher incentives results in higher response rates [36] though a recent study in the Netherlands in people with type 2 diabetes observed that response rates were lower among those offered a 12.5-euro incentive compared to a 7.5 euro incentive [37]. Furthermore, some [38, 39] but not all [37] studies indicate that incentives may improve representativeness of the study sample because incentives have a relatively stronger effect in socio-demographic groups with a relatively lower response rate. Second, we are unable to calculate a response rate because we closed the focus groups once the target sample size was reached. Though not a limitation, we oversampled from certain groups (e.g., women and officers); thus, it is not possible to compare demographic characteristics of our participants to those of the military in general. Furthermore, as a goal of qualitative research is to obtain a variety of perspectives, samples are often not a simple cross-section of the population of interest. This fact is true of participants in this study. Third, opinions about how best to recruit individuals into a research study and compensation may have been impacted by the fact that we recruited the sample by mail and provided the compensation we did. Lastly, we recruited those living relatively close to urban centers, so findings may not generalize to rural Veterans.

## Conclusions

Our study provides practical information to aid investigators in avoiding pitfalls that may hinder recruitment and incorporating information that may help motivate individuals to participate. Many of our findings align with principles for patient-centered outcomes research, which includes trust, honesty, co-learning, transparency, and respect [40]. We encourage researchers to partner with members of their target population to get feedback on recruitment materials and methods. Engaging patients and other stakeholders in the conduct of research can ensure that the research and its results are patient-centered, relevant to the intended users of the research findings, and that the findings can be effectively disseminated [40].

## Additional files

Additional file 1: Focus group guide. Focus group session timing, welcome/ground rules/introductions, and topic introductory text and prompts. (PDF 326 kb)

Additional file 2: Example letter given to participants at focus groups. Example of a contact letter and key study procedure information, annotated in red font to reflect how participants perceieved the letter. (PDF 97 kb)

## Abbreviations

OEF/OIF: Operation Enduring Freedom/Operation Iraqi Freedom
US: United States of America
VA: Department of Veterans Affairs
VHA: Department of Veterans Health Affairs
