# Supplementary material for: How can we get Iraq- and Afghanistan-deployed US Veterans to participate in health-related research? Findings from a national focus group study
Source: BMC Med Res Methodol. 2018 Aug 29;18:88. doi: 10.1186/s12874-018-0546-2 (PMC6114046; doi:10.1186/s12874-018-0546-2)
Supplement: Supplementary file 1 — Focus group guide. Focus group session timing, welcome/ground rules/introductions, and topic introductory text and prompts. (PDF 326 kb) [file 12874_2018_546_MOESM1_ESM.pdf]

TOPIC: **CSP 595: Veterans Research Perceptions and Preferences**
Final 10-21-15

Session Timing	Approx. Time
Welcome/Ground rules/Self-Introductions	10 minutes
Motivations to participate or not participate in research	10 minutes
General Perceptions of Health-related Research with Veterans	15 minutes
Key incentives to increase participation in research	15 minutes
Evaluation of recruiting materials	20 minutes
Data Sharing perceptions and feedback	10 minutes
Suggestions and Wrap-up questions	10 minutes
**Total Time Allotted**	**90 minutes**

WELCOME/GROUND RULES/SELF-INTRODUCTIONS	10 minutes

INTRODUCTION

- Moderator's name, role of moderator in session
- Length of Stay: 90 min
- Looking for diversity in opinions: Nobody will judge your opinions

DISCLOSURES

- Standard disclosures:
- Independent consultant; do not work in the VA
- Audio taping/reasons
- Remind: Informed consent

"RULES"

- Turn off cell phones
- OK to have differing opinions
- No right or wrong answers
- To move conversation along, I may have to interrupt. I just want to make sure that you leave on time!

INTROS

- Moderator will ask the participants to introduce themselves: name, military background, length of service, where are you from? And interesting fact.

**Motivations to Participate or Not Participate in Research 10 min**

Today we are going to be talking a lot about the health-related research that the VA conducts with Veterans. Ultimately, the goals of today's session are to get your feedback and opinions on these research studies and for me to understand what matters most to you so we can improve and have more Veterans participating in research projects.

That said, I am a researcher and not affiliated with the VA. I really only know about research and do not know about the other benefits and services you may have access to as a Veteran. So while I know you may want to tell me about other areas of the VA, I will ask you to try to stay focused on the research studies. That is what I can help them improve. Ok?

We know that everyone has their own reasons for wanting or not wanting to participate in research. Today it would help me if you could tell me what you think about when you get an invitation to participate in a VA research study. And it would also help if you tell me what you think other Veterans may be thinking when they get invited as well.

- If I asked you to take a moment to think of reasons why you wanted to participate in today's focus group, what would you tell me?
- Did any reasons come to mind why you might not want to participate in today's focus group?
- How did it make you feel to be invited to this research project?

**General Perceptions of Health-related Research with Veterans 15 min**

So let's think about Veteran's health-related research studies. These are research studies where you are asked about your health and perhaps have a medical exam or testing:

- Have you been invited to participate in participated in any research?

If yes:

- Why did you decide to participate?
- How often?
- How do you get invited?
- How does it make you feel to be invited to participate?
- What aspect of participating in research do you value most?
- What aspect of participating in research do you value least?
- How did it make you feel to be a part of the study?

If no (invited to participate but decided not to):

- Why did you decide not to participate?

- What matters most when making your decision to participate?
- What do you think matters most to other Veterans?
- Why do you think other Veterans may choose not to participate?

All:

- Do you think Veterans benefit from research? If so, how?
- What do you think the major benefits are to Veterans?
- Have you personally benefited from research with Veterans?
- Are there any issues with conducting research with Veterans?

Perceptions of VA

- If I asked you how you feel about the VA would you say positive, negative or neutral? (show of hands)
- When you think about the health-related studies to that the VA conducts, do you feel better, worse or the same about those as you do about the VA overall. (show of hands).

Key Incentives to Increase Likelihood to Participate 15 min

Let's talk about some specifics:

Invitation Process:
What is the best way for the VA to invite you to participate in research?

- Phone?
- Mail?
- Email?
- What do you like most and least about each way?

- Which is more likely to get your attention and interest?
    - If email isn't an option, what is the next best option to contact you about a research study?

Distance:
Sometimes to participate in a study, you have to travel to get there.
- Is there a distance that impacts your likelihood to participate?
- How many miles are you willing to travel to participate?
- How much time are you willing to spend traveling to participate (minutes/hours)?

Duration of Study Participation: (the amount of time it takes to participate)
How much does the amount of time needed to complete the research study impact your decision to participate?

Imagine that you are being asked to participate in a study that requires filling out questionnaires and one in-person study visit:
- How much time are you willing to spend filling out a questionnaire at home prior to coming in for the study visit?
- This questionnaire may include a medications list, health history form, and log of dates and locations associated with your military service.
- What amount of time seems reasonable to you?
  Probe the following time frames:
    - Up to 30 minutes
    - Up to 1 hour
    - Up to 2 hours
    - Other (less or more)

As I mentioned earlier, this research study would also include an in-person study visit where you would be interviewed and provide some health metrics.

Location:
- The study visit will be conducted at a VA medical center so how do you feel about that?
    - Do you have any issues with visiting the VA medical center to participate in the research?
        - Are there any good things about it being done at a VA medical center?
        - Are there any negative things about it being done at a VA medical center?
            - Parking?
            - If valet, reserved parking options, etc. would that help?

As I said, this research study would also include an in-person study visit where you would be interviewed and provide some health metrics.
The health metrics are things like height, weight and pulmonary function. They have you blow into a machine.

In terms of the in-person visit, are you comfortable with these types of health metrics?

- Is there anything you don't want to do?

The way this study would work:

- Each person will have their own appointment time and will be seen individually by study staff. There won't be a lot of standing in lines and waiting like I know can happen in the military.

Excluding travel time, when you are considering participating in the research study, what amount of time seems reasonable to you for the in-person study visit?

Probe the following time frames:

- Up to 2 hours
- Up to 3 hours
- Up to 4 hours
- Other (less or more)

Excluding travel time, when you are considering participating in the research study, what amount of time seems reasonable to you for the in-person study visit?

Probe the following time frames:

- Up to 2 hours
- Up to 3 hours
- Up to 4 hours
- Other (less or more)

Compensation Models and Delivery Options:

- I know a part of participating in research is being compensated for your time. Which method of compensation do you prefer and why?
    - Probe any preferences or issues with all types
    - Cash
    - Check
    - Debit Card
    - Gift Card (what type of gift card?)

- Does it make a difference if you get the compensation in person when the study visit is complete or if it is mailed to you after the study visit?
    - Why?
    - Does it impact your decision to participate?
- Is there an amount of compensation that seems most fair?
- Do you have an idea about the compensation you think is fair if you think about it as dollars per hour?
- Is there a dollar amount you are looking for as you evaluate whether or not you want to participate?
- Depending on the time and what's involved, most studies offer between $25 and $250 for participation. Research studies that have you fill out a questionnaire at home and mail it in are at the lower end of the scale and those that require pre-paperwork and an in-person visit, compensate more.
    - How do you feel about that?
    - Does it impact your decision to participate?

**Evaluation of Recruiting Materials**	**20 min**

<u>Logos</u> (show on paper – first Dept VA logos then Study logos, separately)

U.S. Department of Veterans Affairs Logos

1 2 3

Study Logos

1

2

3

4

Logo questions by section (VA and Study):

- Do any of these look familiar? Which?
- Which do you like most? Least?
- Which catches your attention?
- Which looks most official?
- Which looks most important?
- If it came on an envelope, which are you most likely to open?
- Which seems to best match what you would expect to see from a VA health-related research study?

Invitation:

- Have you received one in the mail?
- What caught your attention? Anything on the envelop?
  - What information are you looking for when deciding to open it?
- How about the invitation letter?
  - Is there anything that makes you more likely to participate/call?
  - Is there anything that makes you less likely to participate/call?
  - Any advice on what should be mentioned in this letter to increase participation?

Study Descriptions:

- Are there any things you look for in terms of what the study is about and what you would have to do to participate that are important to you?
- Have you seen or read about VA health-related research with Veterans in the past? Were those studies interesting to you? Would you have participated? Why?

Distribute example letter for CSP study.

- Anything about this study look appealing?
- Anything concerning?
- If you got this at home, are you likely to want to participate?
- Why or why not?
- Is there anything they could say in this letter than would make you more likely or interested in participating?

Revised letter probes:

- A reminder -- study is individual not group session:
    - This study would not be a group session like this focus group.
    - Each person will have their own appointment time and will be seen individually by study staff.
    - It will be a very full 2-3-hour study visit (i.e., no standing in line or waiting around). You would complete health and military service questionnaires and some would be filled out yourself, while others would be an interview with a researcher. The surveys are quite comprehensive.

    - How do you feel about that?

Distribute summary of key messages in letter and consent form.

Let's talk about a few of the main things they are trying to tell you in the letter and consent form?

- Anything about this study look appealing?
- Are you likely to participate?
- Anything concerning?
- Is there anything you would want to know more about?

The drug:

- How do you feel about the drug that is mentioned?
- Does it impact your interest in participating?
- Why or why not?
- Any concerns?
- What else do you need to know?

Drug Description:

- The inhaled bronchodilator (drug) that is mentioned in the study description is albuterol, which is used to treat lung diseases such as asthma and chronic obstructive pulmonary disease (COPD).
- The drug is commonly used in pulmonary function testing.
- It is not a study or experimental drug.

- Now that you know more, how do you feel about it?

Data Sharing Perceptions and Feedback	10 min

One of the things the researchers could do is share information and results from the study.

- What types of things would you like to know about yourself and other study participants?
- How do you think study data should be shared with you? (e.g., newsletter?)
- Any concerns about sharing the data?
- Does getting to view some of the study data impact your interest and willingness to participate?

I want to talk to you about some examples of the types of data that could be shared after a study.

What I would like to know is what information would be most valuable to you, and if having this information after the study is complete would make you more likely to participate in the study.

1. Individual level (show example individual report letter and graph. This would provide information about your individual test results and whether they were normal or abnormal results.)
2. Cohort level info (share description – this would provide information about people who were deployed at the same time)
3. Research Study Publications (show example and try not to distribute – these are professional reports that are published in medical and academic journals).

- How valuable are each of these types of information?
- Would it impact your decision to participate in a study?
- Do you think if you got the information, it would impact your decision to participate in future studies?

Suggestions and Wrap-up Questions **10 minutes**

- What do you suggest the VA does to encourage Veterans to participate in health-related research?
- Any interest in the following:
    - Invite Veterans to participate in planning meetings as lay investigators?
    - Would you be willing to refer other Veteran friends for research studies? Once you participated they could give you a card and you could have other Vets call to be included in future studies too.
- If I had to tell them the three things that are most important to get you to interested in participating in health-related research, what would that be?
- DISTRIBUTE and COMPLETE final survey – PLACE IN HIPPA ENVELOPE
