# Supplementary material for: How can we get Iraq- and Afghanistan-deployed US Veterans to participate in health-related research? Findings from a national focus group study
Source: BMC Med Res Methodol. 2018 Aug 29;18:88. doi: 10.1186/s12874-018-0546-2 (PMC6114046; doi:10.1186/s12874-018-0546-2)
Supplement: Supplementary file 2 — Example letter given to participants at focus groups. Example of a contact letter and key study procedure information, annotated in red font to reflect how participants perceieved the letter. (PDF 97 kb) [file 12874_2018_546_MOESM2_ESM.pdf]

VA Cooperative Studies Program

Service and Health During the Iraq and Afghanistan Era (CSP #595)

[SITE NAME]
[SITE ADDRESS]

<Date>

<FirstName LastName>
<MailingAddress>
<City>, <State> <ZipCode>

Annotation in boxes reflect feedback from focus group participants

Dear Mr./Ms. <FirstName LastName>:

Second statement considered vague

The Department of Veterans Affairs (VA) ranks as one of the nation's leaders in health research. This research has significantly contributed to advancements in health care for Veterans and other Americans from every walk of life. These important health care advances are only possible because Veterans volunteered to take part in research projects.

People liked being invited

Purpose of study seems hidden

We are inviting you to take part in a research study called, "Service and Health During the Iraq and Afghanistan Era," sponsored by the VA Cooperative Studies Program (CSP). This research is being done to better understand the health of Veterans who deployed in support of Operation Enduring Freedom (OEF), Operation Iraqi Freedom (OIF), or Operation New Dawn (OND). Your participation may provide information that will improve the lives of others in the future.

You were randomly selected for this study from a list of Department of Defense military personnel who served during the Iraq and Afghanistan era. We are interested in all Veterans of the U.S. Army, Marine Corps, and Air Force who deployed to Afghanistan, Iraq, Kuwait, Djibouti, Qatar, Kyrgyzstan, or the United Arab Emirates between October 1, 2001 and December 31, 2014. It is important for the success of the study that as many Veterans as possible participate. You are eligible to participate regardless of whether or not you receive health care services or disability from the VA.

Meaning of"non-invasive" not clear

Your participation would involve traveling to the [SITE NAME] for a 2 to 3 hour study visit to complete questionnaires and non-invasive tests and measures. Information from this study will also be stored in a subject registry and data repository for future research studies.

Participants noticed the $250 and generally did not recommend moving it up.

Please read the enclosed consent form to learn more about the study and its procedures. If you are interested in participating, please call the study staff toll-free at 1-800-[###]-[####] to arrange your study visit. You will receive $250 upon completion of the study visit to compensate you for your time and effort.

We encourage you to call us at 1-800-[###]-[####] if you have any questions or would like additional information. If you do not want to take part in this study, please return the enclosed Opt-Out Form or call and let us know. If we do not hear from you, we will try to reach you again by mail and by telephone.

Thank you for taking the time to consider participating in this study.

Sincerely,

[SITE PI NAME]
[SITE NAME]
Cooperative Studies Program, Department of Veterans Affairs

Enclosures
